# Identification biomarkers in disease progression of obstructive sleep apnea from children serum based on WGCNA and Mfuzz

**DOI:** 10.3389/fneur.2024.1452507

**Published:** 2024-10-01

**Authors:** Simin Gao, Dan Shan, Yuedi Tang

**Affiliations:** ^1^Department of Otolaryngology-Head and Neck Surgery, West China Hospital, Sichuan University, Chengdu, Sichuan, China; ^2^Department of Otolaryngology-Head and Neck Surgery, Sleep Medicine Center, West China School of Public Health and West China Forth Hospital, Sichuan University, Chengdu, Sichuan, China

**Keywords:** obstructive sleep apnea, diagnostic approach, GSEA, biomarkers, systemic inflammation, prediction model

## Abstract

Obstructive sleep apnea (OSA) syndrome is a prevalent form of respiratory sleep disorder, with an increasing prevalence among children. The consequences of OSA include obesity, diabetes, cardiovascular disease, and neuropsychological diseases. Despite its pervasive impact, a significant proportion of individuals especially children remain unaware that they suffer from OSA. Consequently, there is an urgent need for an accessible diagnostic approach. In this study, we conducted a bioinformatic analysis to identify potential biomarkers from a proteomics dataset comprising serum samples from children with OSA in the progression stage. In the Gene Set Enrichment Analysis (GSEA), we observed that the complement and immune response pathways persisted throughout the development of OSA and could be detected in the early stages. Subsequent to soft clustering and WGCNA analysis, it was revealed that the Hippo pathway, including ITGAL and FERMT3, plays a role in mild OSA. The analysis revealed a significant alteration of the complement and coagulation pathways, including TFPI and MLB2, in moderate OSA. In severe OSA, there was an association between hypoxia and the extracellular matrix (ECM) receptor interaction and collagen binding. In summary, it can be posited that the systemic inflammation may persist throughout the progression of OSA. Furthermore, severe OSA is characterized by abnormal vascular endothelial function, which may be attributed to chronic hypoxia. Finally, four potential biomarkers (ITGAL, TFPI, TTR, ANTXR1) were identified based on LASSO regression, and a prediction model for OSA progression was constructed based on the biomarkers.

## 1 Introduction

Obstructive sleep apnea (OSA) syndrome is regarded as the most prevalent form of respiratory sleep disorder. OSA is characterized by the relaxation of the throat muscles during sleep, which leads to airway obstruction (1, 2). The prevalence of OSA in children is frequently underestimated, with a significant proportion of OSA patients not receiving timely treatment (3). A review of studies published between 2016 and 2023 indicates that the prevalence of OSA in children can reach 12.8 to 20.4%, suggesting an increasing trend over the past decade (4).

The current evidence indicates a strong association between intense local and systemic inflammation and OSA (5, 6). The interruptions in breathing, which are known as apneas and hypopneas, result in periods of hypoxia, which are followed by a return to normal oxygen levels. This fluctuation in oxygen levels triggers the respiratory drive, which ultimately leads to the individual being awakened from sleep (7). A number of studies have indicated that repetitive hypoxia and reoxygenation in OSA are likely to lead to oxidative stress which may play an important role in activating an inflammatory response (8–11). A meta-analysis indicates that a series of inflammatory markers were elevated in individuals with OSA, including C-Reactive Protein (CRP), Tumor Necrosis Factor (TNF)-*α*, Interleukin (IL)-6, IL-8, intercellular cell adhesion molecule (ICAM), Vascular Cell Adhesion Molecule (VCAM), and selectins (12). The concentration of cell-free DNA in serum from OSA patients was found to be elevated, which may indicate cell damage or an acute inflammatory response (13). The biomarkers in the blood have the potential to serve as a means of monitoring the progression of OSA. In addition, nuclear factor kappa B (NF-κB) is locally and systemically activated in children with obstructive sleep apnea syndrome (14, 15). A study provides insights into the expression profiling of miR-27 and let-7 and explores a set of potential target genes of these two miRNAs, CNR1 and CRY2, thus providing hope for the clinical relevance of OSA (16).

Currently, a significant proportion of individuals with OSA remain undiagnosed. The diagnosis of OSA is dependent on polysomnography, but its cost, accessibility, and the need for a skilled medical technician limit its widespread use (17). Therefore, there is a pressing need for biomarkers to facilitate the assessment of OSA. In this study, we employed a bioinformatics approach to investigate potential biomarkers for the progression of OSA. Proteomic data from children’s serum samples across different OSA severity classes were obtained from the ProteomeXchange database (No. PXD032734) (18). It is anticipated that alterations will be observed in blood samples as OSA progresses. In addition, we constructed a model for predicting OSA progression based on these potential biomarkers, providing theoretical support for clinicians’ diagnosis.

## 2 Methods

### 2.1 Data collection and processing

The proteomic data of serum from children with OSA (ID: PXD032734) were downloaded from ProteomeXchange.^1^ The children in the dataset were divided into four subgroups using the apnea-hypopnea index (AHI) classification: AHI < 1 was defined as the control group (Non-OSA), AHI 1–5 was defined as the mild OSA group (Mild-OSA), AHI 6–10 was defined as the moderate OSA group (Moderate-OSA), and AHI > 10 was defined as the severe OSA group (Sever-OSA) (18).

The principle coordinate analysis (PCoA) was employed to reflect the overall expression characteristics of proteins. PCoA and partial least squares discriminant analysis (PLS-DA) were conducted using the SIMCA software (V14.1). The reliability of the PLS-DA model was evaluated through 200 permutations.

### 2.2 Identification of differentially expressed proteins

The R package Limma (v3.48.3) was used to identify differentially expressed proteins (DEPs) between patient subgroups (Mild-OSA, Moderate-OSA, and Severe-OSA) and children in the control group (Non-OSA) respectively. DEPs with a log2 fold change (logFC) of at least 0.263034 were considered to have up-regulated expression, while thoes with a logFC of at most −0.263034 were considered to have down-regulated expression.

### 2.3 Gene set enrichment analysis (GSEA) of OSA subgroups

Gene set enrichment analysis is capable of identifying and analyzing synergistic effects between genes by examining predefined gene sets. The Gene Ontology (GO) and Kyoto Encyclopedia of Genes and Genomes (KEGG) databases were selected as predefined gene sets. The R package clusterProfiler (v4.2.1) was used to perform GSEA on the genes corresponding to DEPs between different subgroups of patients and children in the Non-OSA group. The gene symbols corresponding to DEPs can be identified via the Uniprot database^2^. A positive Normalized Enrichment Score (NES) value in the GSEA results indicates that the gene is upregulated on the pathway in question. Conversely, a negative NES value indicates that the gene is downregulated on that pathway.

### 2.4 Soft clustering of OSA progression-related proteins

To investigate the overall protein expression patterns, a soft clustering method, namely fuzzy c-means clustering was performed using the R package Mfuzz (version 2.48.0). In brief, the mean protein expression value per stage (Non-OSA, Mild-OSA, Moderate-OSA, and Severe-OSA) was initially calculated and subsequently fed to the fuzzy c-means algorithm, which yielded this six serum protein clusters, each with distinct expression patterns.

### 2.5 Weighted gene co-expression network analysis (WGCNA) to screen target proteins

The relationship between OSA serum protein subgroups and gene expression levels was analyzed using the WGCNA package (version 1.71) in R. The optimal *β-*value for subsequent analysis was determined by setting the fitting index of the scale-free distribution topology matrix to 0.9 and subsequently calculating the correlation coefficient between gene modules and subgroups. Subsequently, the correlation coefficient between gene modules and subgroups was calculated.

### 2.6 Protein–protein interaction enrichment for overlap proteins

The overlapping proteins between the DEPs and related serum protein clusters were obtained by drawing a Venn diagram using the R package VennDiagram (v1.6.20). The establishment and enrichment analysis of the protein–protein interaction (PPI) network of overlapping proteins were completed using the STRING online database^3^.

### 2.7 Construction of a prediction model for OSA progression

Lasso regression was used to further screen feature proteins from overlapping proteins as potential biomarkers, and they were incorporated into the multinomial logistic regression model to construct an OSA subgroup prediction model.

### 2.8 Statistical analysis

This study used Pearson’s correlation coefficient as a metric for correlation analysis. A *p*-value of less than 0.05 was deemed statistically significant.

## 3 Results

### 3.1 Protein profiling to distinguish the progression of OSA

**Figure 1:**
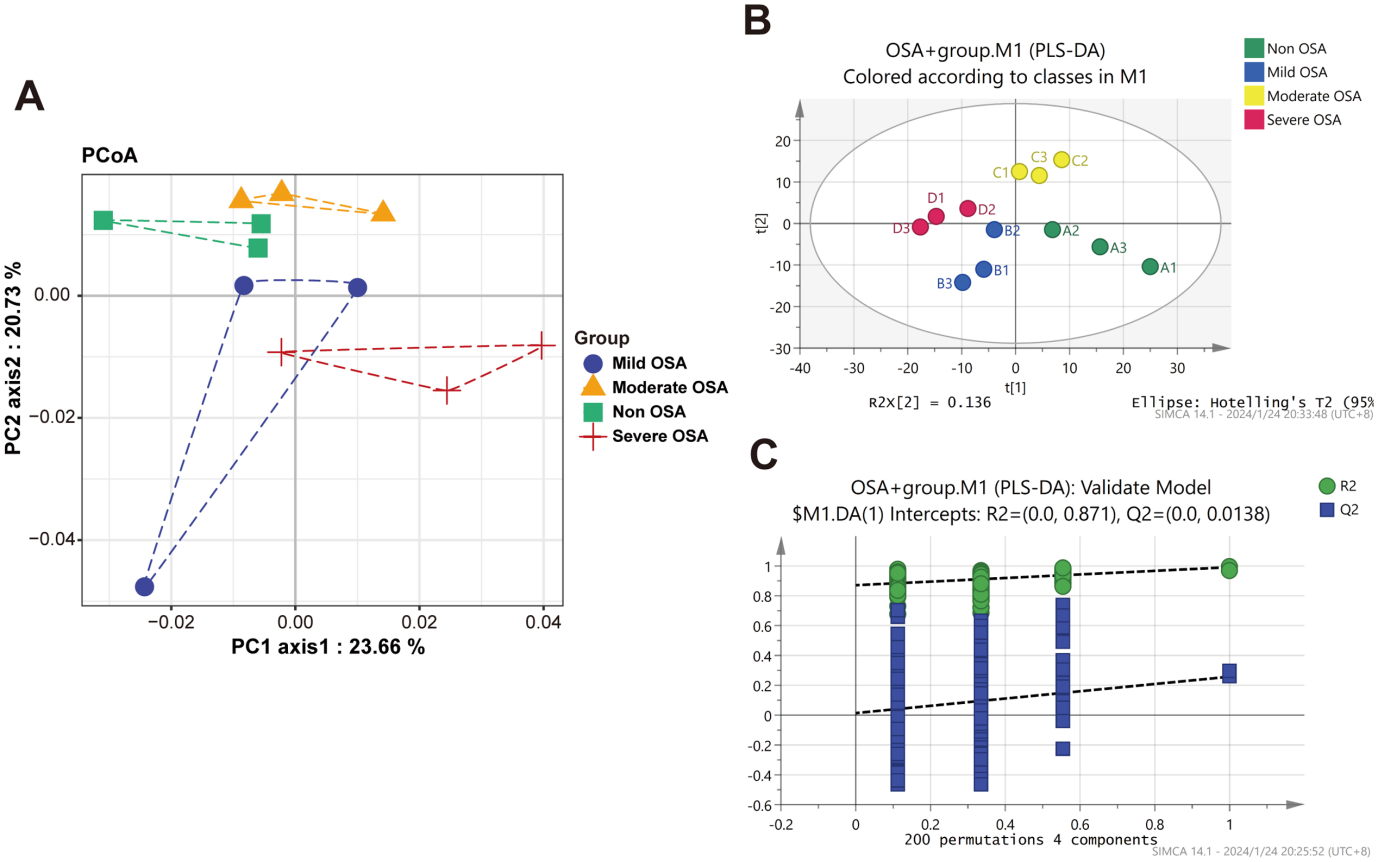
Principle coordinate analysis (PCoA) and OPLS-DA analysis of protein among Non-OSA, Mild-OSA, Moderate-OSA, Severe-OSA. **(A)** PCoA score plot among four groups. **(B)** PLS-DA scatter plot among four groups. **(C)** The results of PLS-DA model replacement test.

The unsupervised PCoA analysis showed a general trend of separation between the Non-OSA group, Mild-OSA group, Moderate-OSA group, and Severe-OSA group (Figure 1A). A supervised PLS-DA model was used to ascertain the extent to which the variables contributed to the observed separation between the four groups (Figure 1B). The model was validated with 200 permutation tests, indicating that the model is robust and not overfitted. This was evidenced by low Q2 values and a negative intercept of the regression line on the *y*-axis (Figure 1C).

### 3.2 DEPs among children in the Non-OSA group and different subgroups

**Figure 2:**
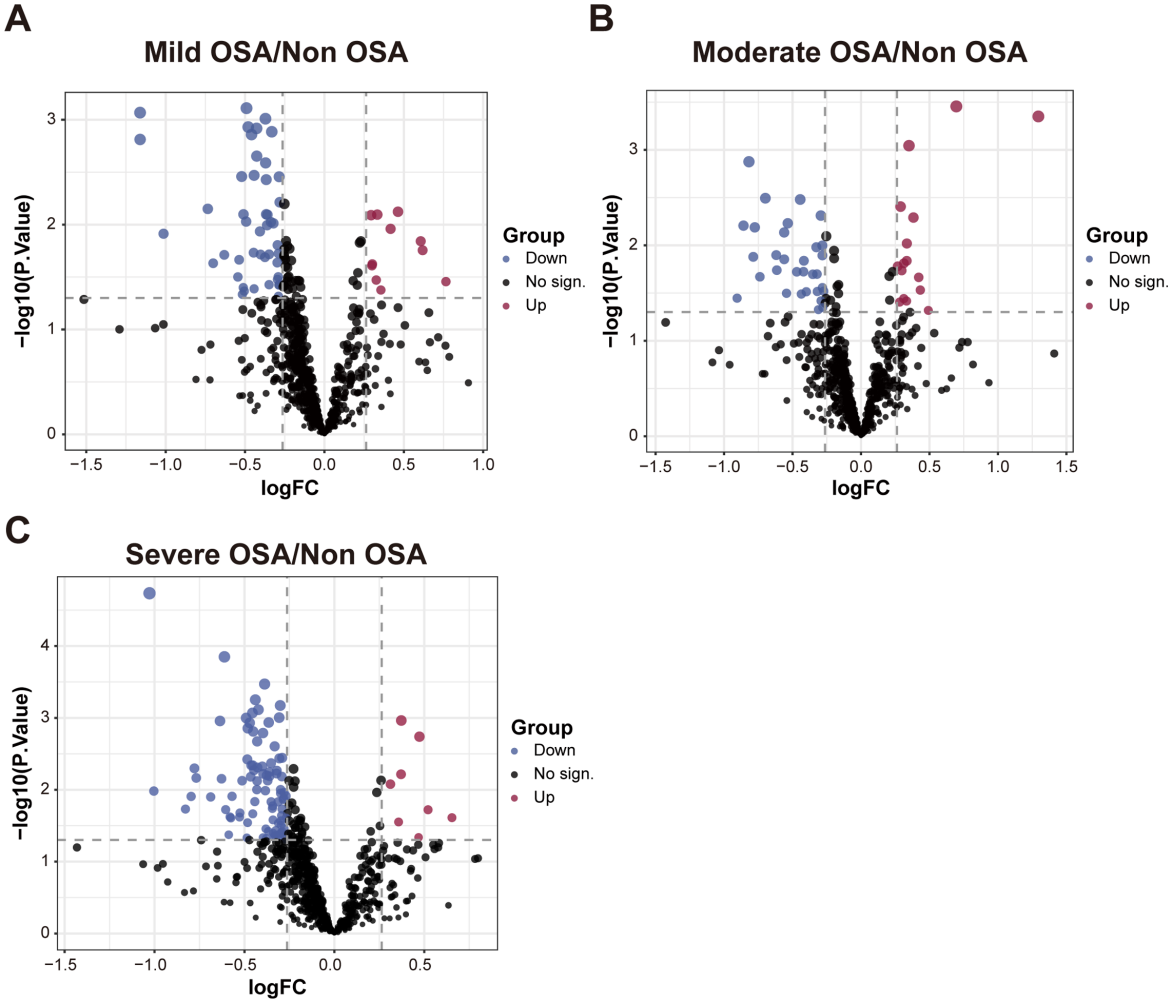
The volcano plot among Non-OSA, Mild-OSA, Moderate-OSA, Severe-OSA. **(A)** The volcano plot shows DEPs between Mild-OSA and Non-OSA. **(B)** The volcano plot shows DEPs between Moderate-OSA and Non-OSA. **(C)** The volcano plot shows DEPs between Severe-OSA and Non-OSA.

The DEPs of serum proteins in normal children and serum proteins of patients with varying degrees of OSA were visualized by drawing volcano plots. The results showed that there were 11 significantly up-regulated DEPs and 46 significantly down-regulated DEPs between the Mild-OSA group and the Non-OSA group (Figure 2A). A comparison between the Moderate-OSA group and the Non-OSA group revealed that 16 DEPs exhibited significant up-regulation, while 31 DEPs demonstrated significant down-regulation (Figure 2B). The number of up-regulated DEPs increased between the Severe-OSA group and the Non-OSA group, with a total of 89 significantly up-regulated DEPs and 8 significantly down-regulated DEPs (Figure 2C). DEPs between each OSA group and Non-OSA group were shown in Supplementary Tables S1–S3.

### 3.3 GSEA results of DEPs in different subgroups

**Figure 3:**
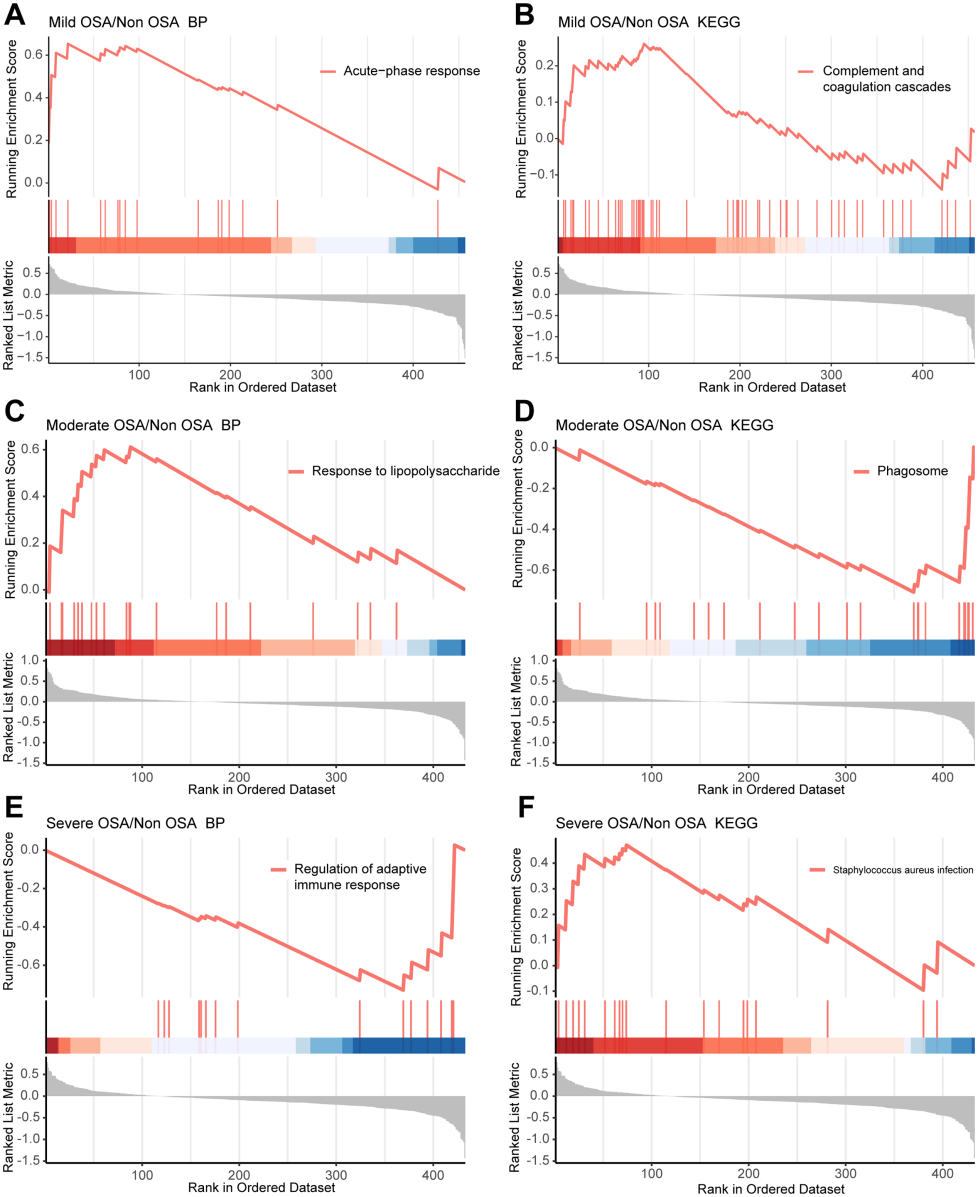
GSEA in Mild-OSA and Severe-OSA. **(A,B)** GSEA plots showed significant pathway in Mild-OSA group. **(C,D)** GSEA plots showed significant pathway in Moderate-OSA. **(E,F)** GSEA plots showed significant pathway in Severe-OSA group.

A GSEA based on GO and KEGG datasets can be employed to investigate the global characteristics of genes in OSA progression and the biological pathways in which they are involved. GSEA based on GO and KEGG datasets can explore the global characteristics of genes in the progression of OSA and the biological pathways they are involved in. Genes within the enriched pathways that changed significantly in GSEA were shown in Supplementary Table S4. In comparison to the Non-OSA group, genes within the complement and coagulation cascade pathways and those involved in acute phase biological processes exhibited a notable up-regulation in the Mild-OSA group and the Non-OSA group (Figures 3A,B). Compared with the Non-OSA group, Moderate-OSA genes were significantly up-regulated in response to lipopolysaccharide and down-regulated in phagosome-related pathways (Figures 3C,D). In addition, compared with the Non-OSA group, genes in the Severe-OSA group were observed to be significantly down-regulated in the immunoglobulin-mediated regulation of adaptive immune responses and significantly up-regulated in *Staphylococcus aureus* infection (Figures 3E,F).

### 3.4 Soft clustering of serum protein expression patterns

**Figure 4:**
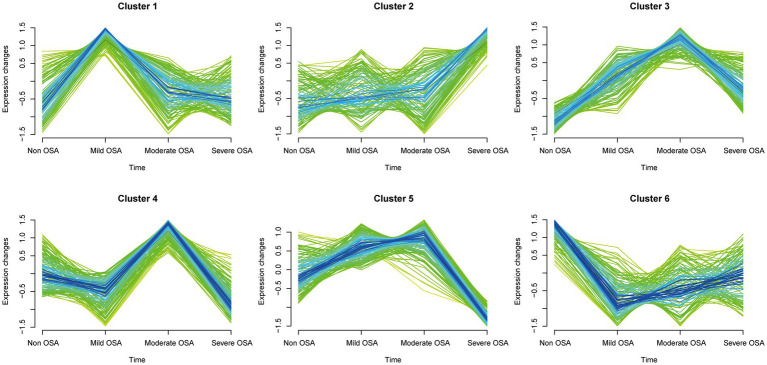
Soft clustering of serum protein expression patterns in progression of OSA. Each mean protein expression values are shown in each broken line. Four groups on the horizontal axis represent the progression of OSA. The six soft clusters exhibit distinct expression patterns of serum proteins across the different stages of OSA.

A total of 752 serum proteins were identified and the longitudinal evolution of the mean expression of proteins across the four periods of OSA was assessed using a fuzzy algorithm. The clustering results were presented in Figure 4. The expression patterns were represented by colored trend lines and characterized by the fluctuation of all proteins in Non-OSA, Mild-OSA, Moderate-OSA and Severe-OSA.

The expression of histones in the Mild-OSA group in both cluster 1 and cluster 6 showed obvious changes. In contrast, the protein expression in the Mild-OSA group in cluster 6 was significantly reduced, while the protein expression in the Moderate-OSA and Severe-OSA groups remained stable, indicating that the proteins in the cluster 6 can reflect mild OSA. Similarly, the peak expression of proteins in the Moderate-OSA group in cluster 4 was observed, and these proteins showed insignificant fluctuations in the Non-OSA, Mild-OSA, and Severe-OSA groups. These observations led to the conclusion that cluster 4 was a more representative cluster for moderate OSA than cluster 3. In cluster 5, as OSA developed, protein expression increased gradually until the Moderate-OAS group, after which it decreased in the Severed-OSA group. Although proteins in cluster 2 also showed a similar trend, the limited number of proteins in cluster 2 rendered it insufficient to reflect severe OSA. Therefore, the clustering proteins may be implicated in the development of severe phenotype in OSA.

### 3.5 WGCNA to investigate the gene cluster related to phenotype

**Figure 5:**
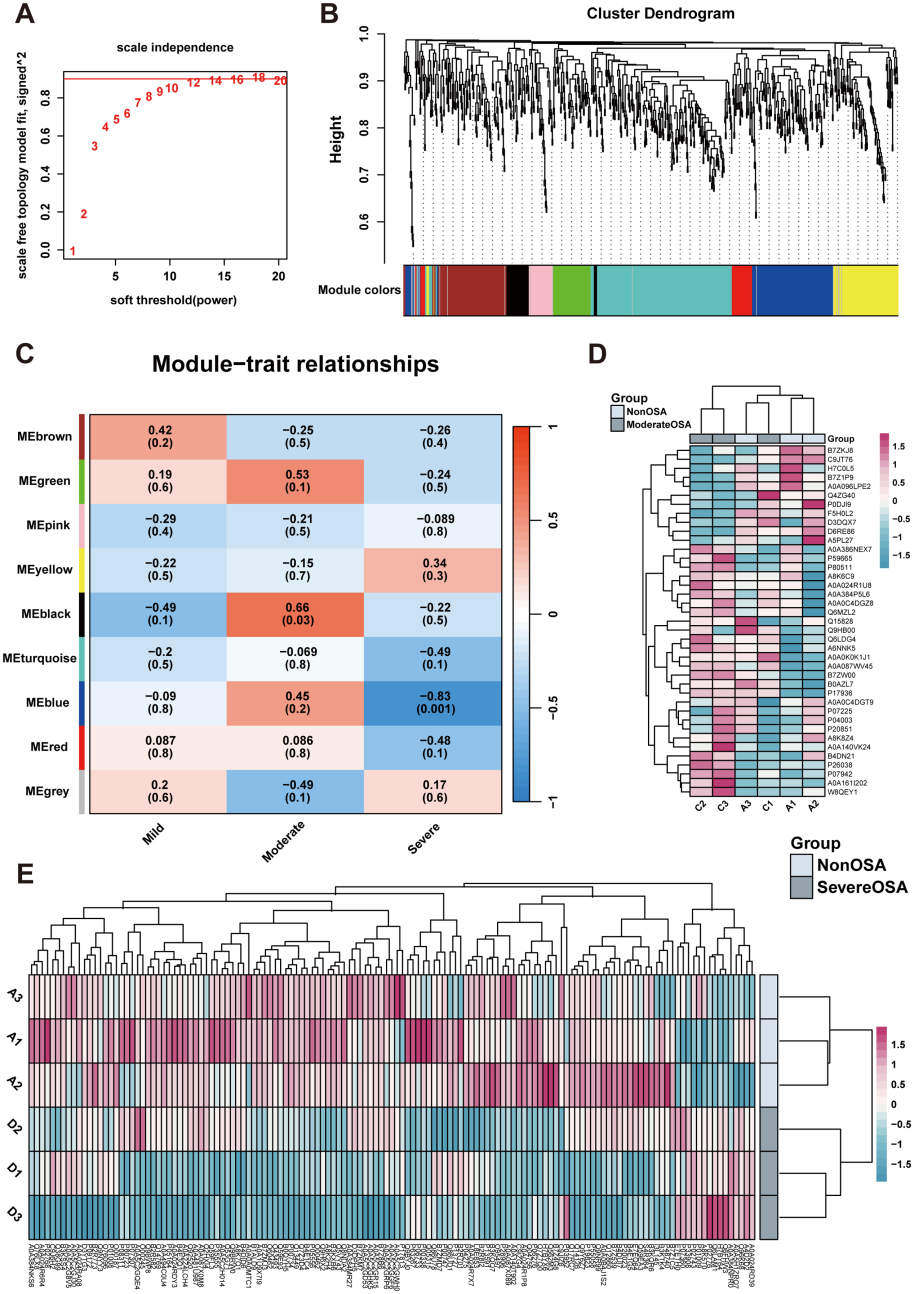
WGCNA analysis. **(A)** Network topology for soft-thresholding powers. **(B)** Proteins dendrogram represents 9 color-coded modules which contains a group of highly correlated proteins. **(C)** Heatmap of the correlation of proteins modules with OSA phenotype. **(D)** The heatmap of proteins expression in MEblue of Moderate-OSA. **(E)** The heatmap of proteins expression in MEblack of Severe-OSA.

When the fitting index of the scale-free distribution topology matrix was set to 0.9, the optimal soft threshold of the network was *β* = 12 (Figure 5A). The co-expression network showed that proteins with similar functions were grouped into one module, with a total of 9 modules identified (Figure 5B). In order to investigate the relationship between proteins within a module and the OSA phenotype, a correlation analysis was conducted. The heatmap of the module-trait relationship showed that the MEblack module exhibited a significant positive correlation with the Moderate-OSA group, while the MEblue module exhibited a significant negative correlation with the Severe OSA group (Figure 5C). The expression profiles of the MEblack and MEblue modules were presented as heatmaps, and the protein expression of these two modules was found to be able to differentiate between the Severe-OSA and Non-OSA groups (Figures 5D,E).

### 3.6 PPIs generated by overlapped DEPs

**Figure 6:**
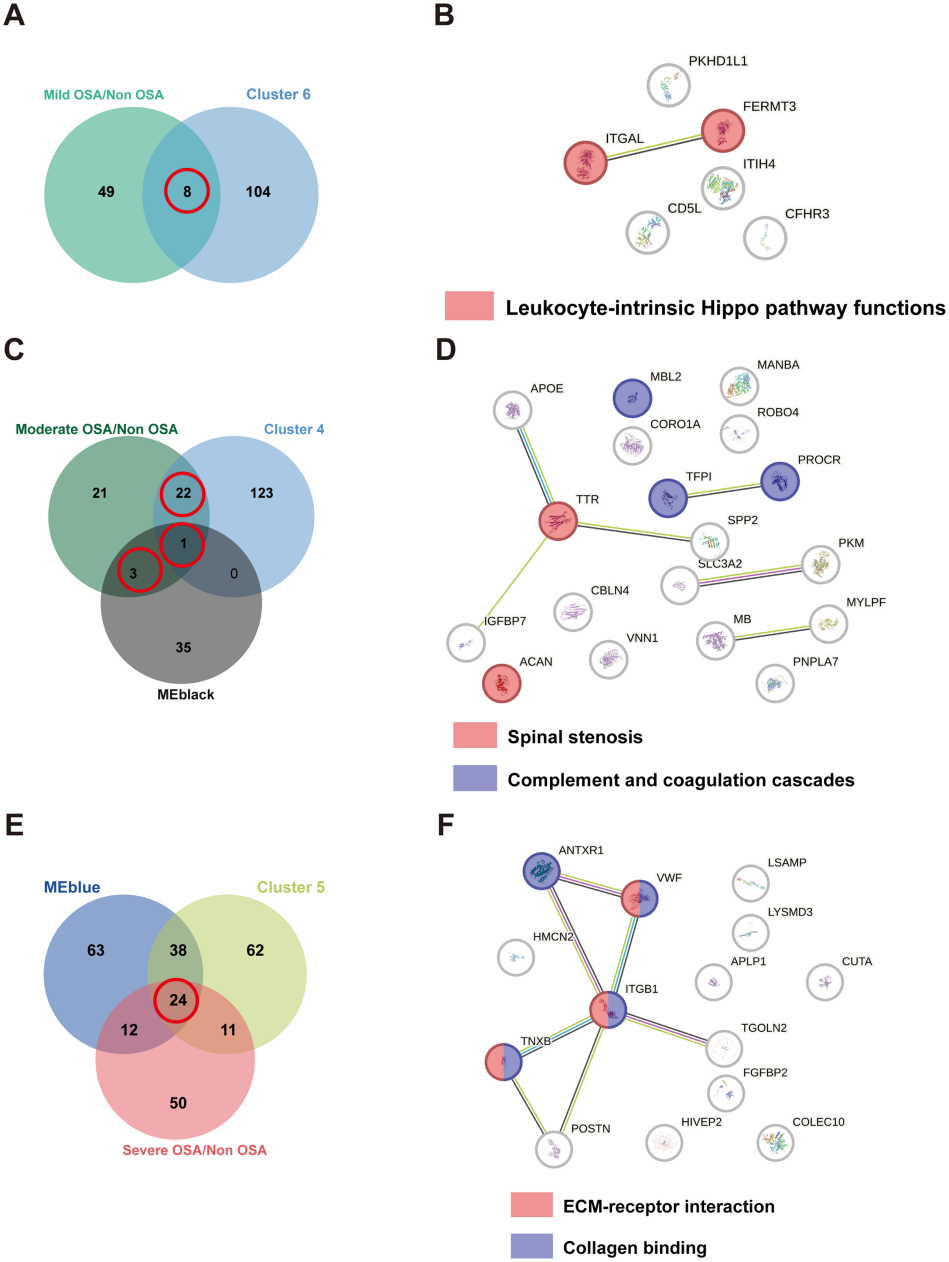
The PPI network of overlapping DEPs. **(A)** The Venn plot of DEPs compared Mild-OSA to Non-OSA and cluster 6. **(B)** The PPI network for overlapping DEPs in Mild-OSA. **(C)** The Venn plot of DEPs compared Moderate-OSA to Non-OSA, MEblack, and cluster 4. **(D)** The PPI network for overlapping DEPs in Moderate-OSA. **(E)** The Venn plot of DEPs compared Severe-OSA to Non-OSA, MEblack and cluster 5. **(F)** The PPI network for overlapping DEPs in Severe-OSA.

The identification of proteins belonging to OSA subgroups would facilitate the investigation of the alterations in biological pathways associated with OSA at each stage. DEPs from soft clusters and modules were used to construct PPI networks and analyze their enriched pathways within the network. Eight DEPs were identified as being shared between cluster 6 and were found to be significantly enriched in leukocyte-intrinsic hippo pathway functions (Figures 6A,B). Twenty-six DEPs were identified as being shared between cluster 4 and MEblack (Figure 6C). These proteins were significantly enriched in pathways associated with spinal stenosis and complement and coagulation cascades pathways (Figure 6D). To identify the most promising target proteins, 24 DEPs were overlapped in cluster 5 and MEblue (Figure 6E). These proteins were enriched in ECM-receptor interaction and collagen binding (Figure 6F).

### 3.7 Prediction model for OSA progression

**Figure 7:**
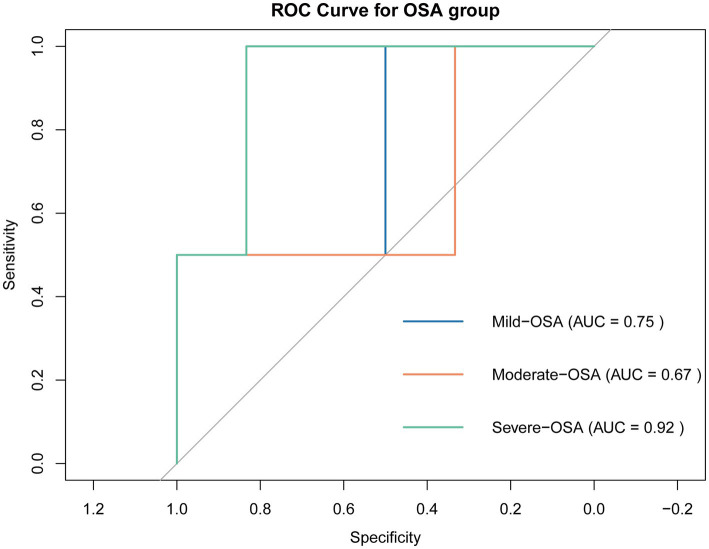
ROC curve of prediction model. Curves of different colors represent the ROC curves of the model when predicting the OSA subgroup.

Based on LASSO regression, we identified 4 potential biomarkers (ITGAL, TFPI, TTR, ANTXR1). We then incorporated them into a multinomial logistic regression model to build a model that can predict OSA progression. As shown in Figure 7, the area under the ROC curve (AUC) of the model in predicting OSA progression (Mild-OSA, Moderate-OSA, and Severe-OSA) was 0.75, 0.67, and 0.92, respectively. This showed that the model has good predictive ability, especially in predicting Severe-OSA. The probability formula for predicting OSA progression by the model is as follows:


$$\begin{array}{l} \text{Probability of Mild}-OSA=4.83410360055754e-05\\ \;+0.126261123884407\times \text{ITGAL}-0.058408460549632\\ \;\times \text{TFPI}+0.000784259406349989\times TTR\\ \;-0.0187933000215171\times \text{ANTXR}1 \end{array}$$

$$\begin{array}{l} \text{Probability of Moderate}-OSA=-4.05161085571589e\\ \;-05-0.023745835903415\times \text{ITGAL}\\ \;-0.0752696562233433\times \text{TFPI}+0.0033212578288373\\ \;\times TTR-0.0233110163382668\times \text{ANTXR}1 \end{array}$$

$$\begin{array}{l} \;\text{Probability of Severe}-OSA=-4.00801914422062e\\ \;-05-0.0201734228318692\times \text{ITGAL}\\ \;-0.00756879802563497\times \text{TFPI}\\ \;+0.00340381285036566\times TTR\\ \;-0.103461527479453\times \text{ANTXR}1 \end{array}$$

## 4 Discussion

In this study, we obtained a serum proteomics data from children with OSA in order to investigate the potential pathways and biomarkers at various stage of disease development. From the prospective of global protein expression, it is evident that there is a considerable degree of variability among the four groups, suggesting that the symptom subtypes may be observed in peripheral fluid. A previous study indicated that a plasma lipidomic profile may serve as a diagnostic fingerprint for severe OSA patients (19). A GSEA revealed that the complement and coagulation cascades pathway plays a pivotal role in primary OSA, when compared to the Non-OSA group. The complement and coagulation cascades, along with their regulatory proteins, are strongly associated with systemic inflammation, creating a potentially devastating amplifying system (20–22). A previous study indicated that the potential role of the complement system, and its activity in OSA are correlated with disease severity (21). Moreover, there is a view that impaired endothelial protection against complement, which is influenced by cholesterol levels, can trigger endothelial inflammation in OSA. Statins can prevent the complement activity to reduce its downstream proinflammatory effects (23). Consequently, the dysregulation of complement and coagulation cascades may be identified in the early stages of OSA.

Furthermore, the expression of genes in the GSEA immune-related pathways was significantly altered in the Mild-OSA, Moderate-OSA, and Severe-OSA groups compared to the Non-OSA group. OSA patients often have cardiopulmonary comorbidities, which trigger an acute phase response during exercise due to altered cardiopulmonary function (24). A previous study employed a blood transcriptome in OSA patients and showed that the genes encoding heavy and light chain immunoglobulins were down-regulated following 12 months of continuous positive airway pressure (CPAP) treatment (25). Both IgA and C3 were found to be significantly up-regulated in children with OSA, suggesting that the complement system was also active (26). Lipopolysaccharide (LPS) can cause the release of inflammatory factors through the innate immune response mediated by Toll-like receptors (TLR), thereby causing inflammation in OSA patients (27). Recurrent hypoxia-reoxygenation cycles in OSA patients can lead to increased oxidative stress, leading to abnormal function of organelles (such as lysosomes), and the phagosome pathway may be inhibited (24, 28). The Severe-OSA group showed significant downregulation of genes in the Regulation of adaptive immune response pathway. This is because the proliferation, differentiation, and effector functions (such as cytotoxicity and helper T cell function) of T cells in OSA patients are inhibited, and the proportion of Treg cells may increase, resulting in an overall suppression of the adaptive immune response (29). Intermittent hypoxia in OSA patients will affect the intestinal environment and blood flow supply, causing intestinal flora imbalance, which will lead to the proliferation of pro-inflammatory bacteria and enhance intestinal and systemic pro-inflammatory responses. This ongoing inflammatory state activates the immune system, causing genes in pathways associated with *S. aureus* to show upregulation (30). These findings implicated that immunity and inflammation were both involved in the progression of OSA.

In soft-clustering analysis, clusters 6, 4, and 5 were used to identify key proteins among the Mild-OSA, Moderate-OSA and Severe-OSA group, respectively. In cluster 6, all the overlapping DEPs exhibited elevated levels in the Mild-OSA group. The leukocyte-intrinsic Hippo pathway, which includes ITGAL and FERMT3, was found to be up-regulated in the Mild-OSA group. The Hippo signaling pathway is regarded as being closely associated with inflammatory responses (31). A transcriptomic analysis of a mouse model of OSA-induced cardiac injury showed that the Hippo pathway was dysregulated in circular RNA compared to control group (32). Similarly, the Hippo pathway was also significantly altered in OSA patients were treated with CPAP (33). ITGAL plays a role in several immune responses. Including leukocyte-endothelial cell interaction, cytotoxic T-cell-mediated killing, and antibody-dependent killing by granulocytes and monocytes (34, 35). FERMT3 plays a role in the integration of platelet adhesion and leukocyte adhesion to endothelial cells (36). The elevated levels of these proteins indicated that the activation of immune and inflammatory responses could be observed in the early stages of peripheral blood in OSA patients.

Moreover, the overlapping DEPs from cluster 4 and MEblack were found to be enriched in spinal stenosis, as well as complement and coagulation cascades, in the Moderate-OSA group compared to the control group. Previous studies have identified a potential association between cervical spine pathologies and sleep apnea (37). The diagnosis of cervical and lumbar stenosis, as well as OSA, can be made in populations with achondroplasia (38–40). Consequently, it seems reasonable to suggest that spinal stenosis and OSA may be considered comorbidities in achondroplasia, and that their relationship requires further investigation. Concurrently, they may exhibit pathological similarities. As previously stated, the complement and coagulation cascades were also significantly enriched in Moderate-OSA. TFPI has the function of inhibiting the extrinsic coagulation pathway. The level of this factor is reduced in the Moderate-OSA group, which may result in the activation of the coagulation cascade in OSA (41). It has been demonstrated that intermittent hypoxia can induce an exacerbation of inflammation in the liver, which is the primary contributor to the production of coagulant and anticoagulant factors (42). Endothelial dysfunction, which can be induced by hypoxic and inflammatory processes in OSA, can concurrently enhance the influence of the extrinsic pathway (43). PROCR is predominantly expressed on the endothelium of large blood vessels, with additional expression on monocytes, and to a lesser degree on neutrophils and eosinophils (44, 45). Our findings indicate that PROCR levels are reduced in the serum of children with OSA. However, elevated urinary EPCR levels have been identified as a potential biomarker for severe adult OSA patients. MBL2 has been identified as a gene that initiates the lectin pathway of the complement system (46). A previous study reported that Italian children with a genotype associated with ‘low MBL’ production exhibited a higher susceptibility to recurrent tonsillitis compared to controls (47). It is well-established that adenotonsillar hypertrophy can result in OSA in children (48). In this study, MBL2 expression was found to be downregulated in the moderate OSA group. Consequently, the dysregulated complement and coagulation cascades can persist from the mild to moderate stages.

The overlapping DEPs from cluster 5 and MEblue were found to be enriched in ECM-receptor interaction and collagen binding in the severe-OSA group compared to the Non-OSA group. Lymphocytes require interaction with glycoproteins and glycosaminoglycans, key constituents of the ECM, to mediate the immune response (49). A study of plasma exosomal microRNA from OSA patients revealed that the top 10 networks highlighted cell-to-cell signaling and interactions, as well as the inflammatory response, including ECM-receptor interaction (50). The vascular basilar membrane is composed of laminin, collagen, fibronectin, and heparin sulfate proteoglycans (51, 52). In the context of hypoxia, the active degradation of the ECM is facilitated by the expression of specific enzymes (53). Moreover, distinct collagen types within the vessel exhibit varying degrees of responsiveness to hypoxic conditions (54, 55). Consequently, prolonged and profound hypoxia in children with OSA may result in the dysregulation of ECM-receptor interactions and collagen binding in the vessel endothelium.

The objective of the present study was to identify potential biomarkers from a proteomics-identified protein profile associated with the progression of OSA in children’s serum. The GSEA analysis indicated that complement and immunity were dysregulated at the global protein expression level and were observed during the early stages of the disease. Following the soft clustering and WGCNA analysis, the MEblue and MEblack modules demonstrated a significant association with moderate and severe OSA, respectively. In the case of mild OSA, ITGAL and FERMT3 were found to be related to the Hippo pathway, indicating that immune and inflammatory responses may occur in the early stages of OSA. Furthermore, complement and coagulation cascades, including MBL2, TFPI, and PROCR have been identified as biomarkers related to phenotypes such as tonsillitis. Moreover, the results indicated that in severe OSA, hypoxia in the vessel may result in altered ECM-receptor interaction and collagen binding in the serum of OSA children. Finally, we used LASSO regression to identify four potential biomarkers (ITGAL, TFPI, TTR, and ANTXR1) among these proteins, and constructed an OSA progression prediction model with accurate predictive performance. The model can calculate the probability of OSA progression in patients based on the expression level of biomarkers, providing theoretical support for helping clinicians make decisions and improving personalized treatment for OSA patients. Nevertheless, further studies and validation in animals and populations are necessary.

## Data Availability

The raw data supporting the conclusions of this article will be made available by the authors, without undue reservation.

## Footnotes

^1^ https://www.proteomexchange.org/

^2^ https://www.uniprot.org/

^3^ https://www.string-db.org/
